# Magnitude of bottle-feeding practice and associated factors among mothers of 0–24 months’ children in Asella town, Oromia region, Ethiopia

**DOI:** 10.1186/s40795-023-00733-w

**Published:** 2023-06-29

**Authors:** Belachew M. Hunde, Ismael K. Sitotaw, Teshome B. Elema

**Affiliations:** 1 Department of Statistics, Arsi University, Asella, Ethiopia; 2 College of Medical and Health Sciences, Department of Public health, Arsi University, Asella, Ethiopia; 3 Clinical Nutrition, Center for Food Science and Nutrition, Arsi University, Asella, Ethiopia

**Keywords:** Bottle feeding practice, Infant and young child feeding, Asella town, Oromia

## Abstract

**Background:**

Since bottle feeding has an impact on the effectiveness of breastfeeding and appropriate supplemental feeding, the World health organization recommends being avoided for infant and early child feeding. Thus, this study aimed to assess the level of the bottle-feeding practice and its associated factors among mothers of 0–24 month’s children in Asella town, Oromia region, Ethiopia.

**Methods:**

Community-based cross-sectional study design was conducted from March 8-April 8, 2022, among a sample of 692 mothers of children aged 0–24 months. A multi-stage sampling technique was used to select the study subjects. Data were collected using a pretested and structured questionnaire by face-to-face interview technique questionnaire. The outcome variable bottle-feeding practice (BFP) was assessed using WHO and UNICEF UK healthy baby initiative BF assessment tools. Binary logistic regression analysis was used to identify the association between explanatory and outcome variables. Adjusted Odds ratio (AOR) with a 95% confidence interval was used to measure the strength of the association and a p-value < 0.05 was used to declare statistical significance.

**Results:**

A total of 692 mothers with mean age and standard deviation (SD) of 31.86 (± 4.87) participated in the study. The prevalence of bottle-feeding practice was 246(35.5% with 95% CI: (31.8, 39.5). Mothers who were government-employed (AOR: 1.64, 95% CI: 1.02, 2.64), mothers who delivered at home (AOR: 3.74, 95% CI: 2.58–5.42), mothers who did not attend postnatal care (AOR: 3.76, 95% CI: 2.60,5.44) and mother who had negative attitude (AOR: 1.94, 95%CI: 1.34,2.8) were significantly associated with bottle feeding practices.

**Conclusion:**

The BFP were higher in the study area when compared with national reports of practices. The occupational status of the mothers, place of delivery, attending postnatal care, and attitude of the mothers were factors that increased bottle-feeding practice in the study area. Strengthening dietary behavioral modification for mothers who have children 0–24 months of the child to practice appropriate feeding is recommended.

## Introduction

Bottle-feeding (BF) is the technique of using a bottle to give a baby any liquid that isn’t breast milk or semi-solid food. The World Health Organization (WHO) classifies exclusive breastfeeding (EBF) as unsatisfactory if it is practiced less frequently than frequently (prevalence < 0.5) [1]. The BF practice of increasing breast milk consumption for newborns over time should be stopped. It is believed that the way milk is typically delivered in bottles makes it more difficult to convert to complete breastfeeding. Throughout the first six months of a baby’s life, formula feeding, and milk consumption are indicators of rapid weight gain [2–6].

WHO recommends that the complete and optimal infant and young child feeding (IYCF) practices were included: on-time initiation of BF within the first hour of birth, EBF, and introducing complementary feeding (CF) from 6 months till year 2 celebration of births or beyond. The IYCF practices are among the contributing factors to alter the nutritional status of children, growth, and body composition of future life in general [7, 8].

Breast milk substitutes are used commonly worldwide with bottle feeding an alternative to breastfeeding which should be avoided due to its impact on complete and optimal breastfeeding and complementary feeding. Moreover, feeding bottles are associated with diarrheal disease morbidity and mortality of under-five as it is difficult to keep clean, especially in low-income and developing countries where sanitation is considered poor. Avoidance of artificial teats or pacifiers is a crucial strategy to promote universal breastfeeding. Exposure and introduction of bottle feeding to infants has been associated with common breastfeeding problems among children. This is due to nipple confusion occurring when infants are exposed to two different feeding methods, bottle, and breast, resulting in the infant refusing to breastfeed, reducing milk production of mothers and unsatisfactory breastfeeding [7–11].

Milk and other liquids, including semi-solid cereals when diluted with water or milk, are frequently given in the bottle. Because of a lack of access to clean water, sanitation, and hygiene as well as a high rate of illiteracy among mothers and guardians, the negative impacts of BFP are especially severe in low-income settings and developing countries. The level of unsuitable and/or low-quality bottles practices and treats further aggravates the situation in developing countries. Bottle contamination and bottle-feeding risks include more than just milk that has been over-diluted; they are also a key contributor to incorrect feeding, which can result in a variety of malnutrition types and stunted growth. Moreover, it made them more susceptible to gastrointestinal tract (GIT) infections, ear infections, allergic tendencies, and dental cavities, as well as other nutrition-related diseases including diarrhea [11].

Studies show that bottle feeding has an impact on a child’s oral-facial development. Long-term bottle feeding raises the risk of posterior cross bite and an open bite. Since it is difficult to clean the bottle teats and because of confusion caused by varied feeding practices, feeding infants using a bottle with a nipple is a contributing factor in respiratory tract infections (RTI) and malnutrition [12]. Infants who are bottle-fed are forced to empty the bottle by their parents or caregivers, exposing them to childhood obesity and diabetes mellitus and increasing their risk of developing gastrointestinal (GI) infections compared to children who are breastfed exclusively [13–15].

The incidence of malnutrition rises sharply during the period from 6 months to 23 months of age in most countries, and the deficits acquired at this age are difficult to compensate for later in life. Malnutrition subsequently leads to morbidity, low cognitive ability, and mortality in infants and young children. It has been responsible, directly, or indirectly, for 60.0% of deaths annually among children under-five in the world and over two-thirds of these deaths are often associated with inappropriate feeding practices [16].

Lack of appropriate child-feeding practices highly contributes to infant mortality and morbidity due to childhood malnutrition and diarrheal diseases which are the major contributors to infant and child death. Globally, an estimated 159 million children under 5 years of age were stunted, but this burden is not evenly distributed around the world, Sub-Saharan Africa (40%) and South Asia (39%) are home to three-fourths of the world’s stunted children [16, 17].

Currently, women are increasingly engaging in their own work leaving a child for caretaker by providing commercial or readymade milk powder to feed the child using a bottle. Even though a couple of studies are made on feeding practice most of them focus on exclusive feeding and complementary feeding, but it is not enough and needs continuous assessment and recommendation to change the practice. There is no recent research done on the prevalence of bottle feeding and associated factors in Asella town and there is a need to know the prevalence of bottle feeding in the town. The purpose of this study is to determine the magnitude of bottle feeding and identify the factors associated with bottle feeding practice in Asella town in order to obtain the baseline data that will help the concerned bodies to plan and implement feasible interventions to alleviate the problems.

## Methods

### Study area and period

This study was conducted in Asella town, Oromia regional state, Ethiopia, from March 8-April 8, 2022. Asella town is located in the Oromia region and has a distance of about 159 km and 75 km from Addis Ababa and Adama respectively in the southeast direction. The town is situated at latitude and longitude of 7.57^o^N and 39.7^o^E, respectively, with an elevation of 2430 m above sea level. As evidenced by the health office of the town shown, the estimated total population is 113,445. Of these 25,105 are mothers of reproductive age groups and children less than five years of age are 18,639 among which 3,652 are between age 0–12 months and 6,466 is between 0 and 24 months age. The town has 8 kebeles (the smallest administrative unit in Ethiopia), 1 government hospital, 2 government health centers, 8 health posts, 2 private hospitals, 14 private clinics, drug stores, and 13 Pharmacies. The total population in Asella town is 113,445 of whom 57,290 are male and 56,155 are females in 2022 [18].

### Study design

The community-based cross-sectional study design was used.

### Source population

All mothers or caregivers in Asella town who had young children aged 0 to 24 months.

### Study population

All mothers or caregivers who resided in a selected kebele of Asella town, and were reachable during data collection, and had children ages 0 to 24 months.

### Inclusion and exclusion criteria

All mothers older than 18 years who had children 0–24 months of age who have been living in area for more than six months were included, but Mothers/caregivers who did not have children or sick children, and who had children older than 24 months were not included in the study.

### Sample size determination

Sample size for the first objective: Sample size was determined by using a single population proportion formula by considering the proportion of bottle-feeding practice was 19.6% [19] with the assumptions of a 95% of confidence level, 3.8% margins of error. A total of 692 samples of data respondents were used in the study, with design effect 1.5 and 10% non-response rate factored into the produced sample.

### Operational definitions

#### Bottle feeding practice (BF)

The outcome variable ‘BF’ is defined as drinking anything from a bottle with a nipple once or more time [8]and was expressed as a dichotomous variable with category 1 for children who drank anything from a bottle and Category 0 for children who did not.

#### Good knowledge

If the mothers correctly answered half and above knowledge questions [20].

#### Positive attitude

From six attitudes measuring questions those who score above mean.

#### Negative attitude

From six attitudes measuring questions those participants who score below the mean [21].

### Sampling procedure

A multi-stage sampling technique was used to select the study subjects. From a total of eight kebeles, four kebeles are selected using a simple random sampling method. The list of mothers who had children 0–24 months was obtained from a health post. The sample size was allocated proportionally among selected kebeles. A systematic sampling technique was used to select households every five intervals with an eligible participant. For households with more than one eligible study subject, only one participant was selected using the lottery method. When the participant was not available at the time of data collection three repeated visits were made.

### Data collection procedure and tool

Data was collected through face-to-face interviews using a pre-tested structured Afan Oromo and Amharic version questionnaire. The questionnaire was adapted from joint report of [22] and by reviewing the literature of similar studies and modified to suit the setting to collect child bottle-feeding practice information accordingly. The main sections of the questionnaire consisted of socio-demographic characteristics, knowledge, attitude, and bottle-feeding practice. Using the KAP accepted evaluation instrument, researchers evaluated mothers’ and caregivers’ knowledge of and attitudes toward bottle feeding techniques. Based on the results of the knowledge evaluation instrument, the knowledge is categorized as either good or bad; it is considered good if the mothers correctly answered more than half of the questions regarding the established bottle-feeding methods. Similar classification criteria are used for having a positive attitude which defined as the mothers who have good attitude for cleaning of bottles. Training was given to data collectors and a supervisor for three days. Data was collected by five trained clinical nurses and 2 public health officers.

### Data quality assurance and validity/reliability

For validity and reliability, we used the validated questionnaire and data collection method from previously published comparable studies [13–15]. A pre-test was done by selecting 5% of mothers/ caretakers who had children 0–24 months of age in Iteya town, near the study area and the necessary correction was taken accordingly. The questionnaire was created in English, translated into the local community language that is most often spoken in Ethiopia (Afan Oromo and Amharic), and then translated back into English in cooperation with a translation professional to ensure consistency. The training was given to data collectors and supervisors for one day. The collected data were checked for completeness and clarity by the principal investigator daily. Data cleaning and cross-checking were done before analysis.

### Data processing and analysis

The data were coded, entered, and exported from Epi-Data version 4.6 to Stata version 16 for analysis. For each variable, a descriptive analysis was conducted. In order to choose potential variables for multivariate analysis, a binary logistic regression model was used in a bivariate study. To reduce the impact of confounders, the bivariate analysis’s variables with p-values 0.25 were transferred to the multivariate analysis. In the final model, variables were judged to have a significant connection between the outcome and the explanatory factors if they had a P-value 0.05 with adjusted odds ratios, together with 95% confidence intervals. Multi-collinearity was checked to test correlation among predictor variables at Variance inflation factor less than 2 and The Hosmer and Lemeshow goodness of fit test was used to determine whether the model adequately describes the data. Finally, the results were presented in the form of tables and texts based on the obtained data.

### Result

#### Socio-economic and demographic characteristics

A total of 692 respondents were included in the study giving a response rate of 100%. The mean age of the mothers and child was 31.86 (± 4.87) years and 10.82 months with ± 6.92 SD respectively. The majority of 202 (90.9%) mothers were married. Regarding occupational status 318(46%) of mothers were housewives. Two hundred fifteen (31.1%) of respondents had primary education (Table 1).

**Table 1:** Socio-demographic characteristics of the respondents, in Asella town, Ethiopia, 2022

Variables(n = 692)	Categories	Frequency	Percent
Mothers’ age	20–24	51	7.4
	25–29	183	26.4
	30–34	226	32.7
	35 and above	232	33.5
Marital Status	Single	9	1.3
	Married	629	90.9
	Divorced and widowed	54	7.8
Religion	Muslim	265	38.3
	Orthodoxy	222	32.1
	Protestant	125	18.1
	Wakefata	70	10.1
	Catholic	10	1.4
Mothers’ education	No formal education	117	16.9
	Primary education	215	31.1
	Secondary and above	360	52
Mother’s Occupation	Housewives	318	46
	Employee	135	19.4
	Merchant	105	15.2
	Daily laborer	54	7.8
	Farmer	81	11.7
Child age in months	0–5	200	28.9
	6–11	185	26.7
	12–23	307	44.4

### Obstetric and Health care service-related characteristics of the respondent

The majority of participants 656 (94.8%) had a history of ANC follow up and more than half of 388 (56.1%) participants had a history of follow-up up. More than three fourth 509 (78.1%) respondents received advice on the advantages of breastfeeding during ANC follow-up. Four hundred fifty-eight (66.2%) were given birth at a health institution (Table 2).

**Table 2:** Obstetrics and health service condition of the respondents, in Asella town, Ethiopia, 2022

Variables	Categories	Frequency (n)	Percent (%)
Parity	One	176	25.4
	Two	171	24.7
	Three	110	15.9
	Four and above	235	34.0
ANC	Yes	656	94.8
	No	36	5.2
Received counseling on IYCF	Yes	509	78.1
	No	143	21.9
Place of birth	Health Facility	458	66.2
	Home	234	33.8
PNC	Yes	388	56.1
	No	304	43.9
Counseled on bottle feeding practice	Yes No	330 292	53.1 46.8

### Knowledge and attitude of the respondent

Of the 692 participants of the study used to assess bottle feeding using KAP assessment tools, 404 (58.4%) participants had good knowledge and 417(60.3%) respondents had a positive attitude as presented in Table 3.

**Table 3:** Knowledge and attitude of the respondents on bottle feeding practice, in Asella town, Ethiopia, 2022

Variables		Frequency (n)	Percent (%)
Knowledge	Good knowledge	404	58.4
	Poor knowledge	288	41.6
Attitude	Positive attitude	417	60.3
	Negative attitude	275	39.7

### Magnitude of the bottle-feeding practices

The overall magnitude of the bottle-feeding practice in the study area was 246 out of 692 (35.5%, 95% CI: 31.8, 39.5). Among 200 children aged 0–5 months, 60(30%) children were practicing bottle feeding, among 185 children aged 6–11 months 74(40%) were practicing bottle feeding and out of 307 children aged 12–24 months 112(36.5%) children were practicing bottle feeding. Among respondents who bottle-fed their infants/children, 140 (56.9%) used cow milk and 106 (43.1%) used infant formula. According to the assessment, 86 out of 692 (34.8%) of mothers did not practice BF because of being too busy with employment outside the home, as shown in Table 4 below.

**Table 4:** Bottle feeding practice among study participants, in Asella town, Ethiopia, 2022

Variables	Categories	Frequency (n)	Percent (%)
Bottle feeding child	Yes	246	35.5
	No	446	64.5
Reason to start bottle feeding	Mother busy	90	36.6
	Good to promote growth	53	21.5
	Convenient	37	15.1
	Insufficient breast milk	35	14.2
	Mothers’ illness	31	12.6
Fluid offered with the bottle feeding	Cow milk	140	56.9
	Formula milk	106	43.1
What frequency of bottle feeding per day?	Two	20	8.1
	Three	58	23.6
	Four	71	28.9
	Five and above	97	39.4
How do you keep the bottle clean?	Boiling	102	41.5
	Rising with water and soap	94	38.2
	Only rising the water	50	20.3

### Risk factors of bottle-feeding practice

Bivariable analysis was computed and variables with p-value < 0.25 were candidates for multivariable analysis. Accordingly, the occupational status of the mother, children’s age, health education/counseling on IYCF, and Post-natal care (PNC) follow-up were significantly associated variables with Bottle feeding practice.

Regarding occupational status, mothers who were employed were 1.6 times more likely to practice bottle feeding than housewives mothers (AOR: 1.64, 95% CI: 1.02, 2.64). Similarly, mothers who were in daily laborer were 2.67 times more likely to practice bottle feeding than housewives mothers (AOR: 2.67, 95% CI: 1.34, 5.31). Mothers who delivered at home were 3.74 times more likely to practice bottle feeding than women who delivered at a health facility (AOR: 3.74, 95% CI: 2.58,5.42). Mothers who did not attend PNC follow-up were 3.76 times more likely to practice bottle feeding than mothers who had attended PNC follow-up (AOR: 3.76, 95%CI: 2.60,5.44). Mothers who had negative attitudes were 1.94 times more likely to practice bottle feeding than positive attitude women (AOR: 1.94, 95%CI: 1.34, 2.8) Table 5.

**Table 5:** Factors associated with bottle feeding practice in Asella town, 2022

		Bottle feeding practice		
Variables(n = 692)		Yes n (%)	No n (%)	AOR (95%CI)
Mother occupational status	Government employee	61(45.5)	73(54.5)	1.64(1.02,2.64) *
	Farmer	29(35.8)	52(64.8)	1.35(0.76,2.41)
	Merchant	35(33.3)	70(66.7)	0.82(0.47,1.42)
	Daily laborer	33(61.1)	21(38.9)	2.67(1.34,5.31) **
	Housewives	88(27.7)	230(72.3)	1.00
Child age	0–5 month	60(30)	140(70)	1.00
	6–11	74(40)	111(60)	1.04(0.67,1.62)
	12–24	112(36.5)	195(63.5)	1.10(0.71,1.70)
ANC service received for current child	Yes	228(34.8)	428(65.2)	1.00
	No	18(50)	18(50)	1.67(0.73,3.8)
Place of delivery of the current child	Health facility	118(25.7)	340(74.3)	1.00
	Home	128(54.7)	106(45.3)	**3.74(2.58,5.42)** **
PNC	Yes	82(21.1)	306(78.9)	1.00
	No	164(53.9)	140(46.1)	**3.76(2.60,5.44)** **
Attitude status	Positive attitude	118(28.3)	299(71.7)	1.00
	Negative attitude	128(46.5)	147(53.5)	**1.94(1.34,2.8)** **

∗Statistically significant at *p* < 0.05; ∗∗ Statistically significant at *p* < 0.001,1 = used as reference.

## Discussion

The purpose of this study was to evaluate the magnitude of bottle-feeding practice (BFP) and related variables among infants under the age of two in Asella town. Compared to national data, the prevalence of bottle-feeding in the current study was somewhat higher [23]. There was evidence that the practice of bottle feeding was influenced by the mother’s occupation, the setting of delivery, her attendance at postnatal care (PNC), and her attitude.

The magnitude of bottle feeding in the current study was similar to the studies conducted in Bangladesh (37%) [24] and Nigeria (35%) [25]. But it is relatively higher compared to studies done in Uganda (10%) [26], in Ethiopian mini-demographic health survey (EMDHS) (9%) [23] in Holota town, Ethiopia (19.6%) [27], and in Shashemene town, Ethiopia (20.9%) [28]. This discrepancy may result from differences in study settings, assessment of sample sizes, and time intervals between investigations. The socioeconomic status differences between Ethiopia and Uganda, cultural customs, the availability of child food items, and nutrition action interventions from the ministry of health-to-health professionals, such as the implementation of health extension programs in Ethiopia, all have an impact on the proper feeding practices. However, the BFP’s size is slightly lower than that of a prior study carried out in Addis Abeba (85.8%) [29] and Agaro town (93%) [30]. The possible explanation could be due to the variation in sample size and amount of mother awareness. Also, it’s possible that the distinction between research locations is determined by the quality of the health care provided, the length of time between the study period and the socioeconomic and non-governmental organizations’ support, as well as other factors. The observed discrepancy may be a result of the study area’s inadequate health care counseling services on IYCF, especially bottle feeding.

In terms of employment status, women who worked, and mothers who were housewives result is consistent with research from Uganda [26] and Jima zone Agaro town [30]. This may be because women who work in government and daily laborer are frequently away from the home. Also, this can be the case because moms of housewives understand nutrition education better than women who work in government.

The results of the study done in Jimma Agaro town, which compared mothers who gave birth at home to those who did so in a medical institution, are consistent with the delivery locations variation of this study [30]. This could be as a result of mothers who gave birth in a medical facility possibly receiving appropriate child-feeding guidance from their medical professionals. This may be an effective method for enhancing child feeding procedures. A study carried out in South Sudan [20] and Holota town [19] provided support for the study’s findings about the attendance of prenatal care such as PNC follow-up. It might be because mothers who underwent PNC follow-up had the opportunity to receive health care provider counseling and instruction regarding child feeding practices. Whereas, if mothers/caregivers had a negative attitude, they would be more likely to bottle-feed their infants than mothers with a positive attitude, according to a study done in Addis Ababa [14]. The women who had a positive attitude and who practiced bottle feeding more might have had a greater probability of doing so since they were more knowledgeable about the IYCF components and had a better comprehension of the issue.

### The limitations of the study

Due to the nature of the study, we were unable to evaluate the observational investigation of BFP in terms of how well it adhered to the bottle-feeding guidelines, including preparation techniques, fluid composition and proportion, sterilizing procedures, and equipment cleaning mechanisms. The study used cross-sectional study designs which do not show cause and effect relationship.

## Conclusion

This study finding showed that the prevalence of bottle-feeding practice was higher in the study area when compared with national prevalence. Mothers whose occupational status were government employees, mothers who delivered at home, mothers who did not attend PNC follow up and mothers who had negative attitude were found to be significantly associated with bottle-feeding practice. We recommend that health offices and health centers in Asella town should strengthen health professionals to counsel mothers during ANC and PNC about feeding practices. Health extensions and health workers should improve the attitude of mothers on bottle feeding by counseling and health education programs on IYCF and health risk of bottle-feeding practice for infants in the study area.

## Data Availability

The dataset used for this study is available from the corresponding author upon reasonable request.

## List of abbreviations

ANC: Antenatal care
AOR: Adjusted Odds Ratio
BF: Bottle Feeding
BFP: Bottle feeding practices
CF: Complementary Feeding
EBF: Exclusive breast feeding
EMDHS: Ethiopian mini-demographic health survey
GI: Gastrointestinal
GIT: Gastrointestinal Tract
IYCF: Infant and Young Child Feeding
PNC: Postnatal care
RTI: Respiratory Tract Infection
SD: Standard Deviation
UNICEF: United Nations Children’s Fund
WHO: World Health Organization
